# Optimizing FeSiCr-Based Soft Magnetic Composites Using the Deionized Water as the Phosphating Solvent

**DOI:** 10.3390/ma17071631

**Published:** 2024-04-02

**Authors:** Xiangdong Li, Hongya Yu, Hongxiang Wang, Tongxin Yuan, Zhongwu Liu

**Affiliations:** 1 School of Materials Science and Engineering, South China University of Technology, Guangzhou 510640, China; lixiangdong1998@163.com (X.L.); 202221022249@mail.scut.edu.cn (H.W.); tongxin_yuan1997@163.com (T.Y.); 2 South China Institute of Collaborative Innovation, Dongguan 523808, China; 3 Dongguan Mentech Optical & Magnetic Co., Ltd., Dongguan 523330, China

**Keywords:** FeSiCr alloy, soft magnetic composite (SMCs), phosphating solvent, insulating coatings, magnetic properties, corrosion resistance

## Abstract

To prepare a soft magnetic powder core, the magnetic powder surface has to be insulated by phosphating treatment. Organic chemicals such as ethanol and acetone are generally used as solvents for phosphoric acid, which may cause serious environmental problems. This work proposed deionized water as the environmentally friendly phosphating solvent for FeSiCr powder. The soft magnetic composites (SMCs) were prepared using phosphoric acid for inorganic coating and modified silicon polymer for organic coating. The effect of different phosphating solvents, including deionized water, ethanol, and acetone, on the structure and magnetic properties of SMCs were investigated. It is found that the solvent affects the phosphating solution’s stability and the phosphoric acid’s ionization. The phosphoric acid is more stable in deionized water than in ethanol and acetone. The phosphating reaction in deionized water is also more stable in deionized water, resulting in a dense phosphate coating on the particle surface. The effects of phosphoric acid concentration and temperature on the magnetic properties of FeSiCr-based SMCs were further studied. With the increase in phosphoric acid concentration and temperature, the magnetic permeability and saturation magnetization of the powder core decrease, and the core loss decreases, followed by an increase. The optimized combination of properties was obtained for the SMCs phosphated with 0.2 wt.% phosphoric acid in deionized water at 35 °C, including a high effective permeability *μ_e_* of 25.7, high quality factor *Q* of 80.2, low core loss *P_cv_* of 709.5 mW/cm^3^ measured at 0.05 T @ 100 kHz, and high withstanding voltage of 276 V, due to the formation of uniform and dense insulating coating layers. In addition, the SMCs prepared with phosphated powder show good corrosion resistance. The anti-corrosion properties of the SMCs using deionized water as a phosphating solvent are better than those using ethanol and acetone.

## 1. Introduction

Soft magnetic composites (SMCs) made of magnetic particles coated with organic or inorganic insulating layers have been widely used to prepare electronic components and devices, such as electric inductors, adapters, transformers, and motors [1]. FeSiCr-based SMCs have attracted much attention due to their high saturation magnetization and low power loss. FeSiCr-based SMCs are being developed to enhance soft magnetic properties and environmental reliability to further meet the higher requirements of low voltage and high current [2,3,4,5].

Magnetic core loss is one of the most critical parameters of SMCs for high-frequency applications, consisting of hysteresis loss [6], eddy current loss, and residual loss [7]. In the MHz frequency, the eddy current loss of SMCs is dominant, dramatically increasing with increasing frequency [8]. The eddy current loss is mainly determined by the resistivity of the material. In SMCs, the eddy current loss can be categorized into inter-particle and intra-particle eddy current loss [9]. The intra-particle eddy current loss is unavoidable for the alloy powder with a defined size and shape, whereas the inter-particle eddy current loss can be minimized by the effective insulation between the neighboring particles [10]. Therefore, the insulation coating process is essential for the preparation of SMCs. There are two types of insulation coating for FeSiCr-based SMCs: organic and inorganic. Epoxy resin, silicone resin, and polyimide resin are generally used as the organic coating [11], while phosphides, SiO_2_, ZrO_2_, Al_2_O_3_, ferrite, etc., can be employed as inorganic coating [12,13,14]. The organic coating materials facilitate the formability of the powder core during compaction, and the inorganic coating materials mainly provide high resistivity to reduce the eddy current losses of SMCs at high frequencies [15]. Currently, the passivation of powder particle surfaces using phosphoric acid solution is the most commonly used method for inorganic coating. The phosphating process mainly consists of two steps: the ionization of phosphoric acid in the solution and the formation of phosphate by the combination of free iron ions (Fe^2+^ and Fe^3+^) and ionized phosphate ions. The quality and properties of the coating are mainly controlled by process parameters such as phosphoric acid concentration, temperature, and time [16,17,18]. The uniform and dense phosphating coating on the particles surface can not only improve the corrosion resistance, but also increase the resistivity and reduce the eddy current loss at high frequency [19]. Moreover, it can also enhance the breakdown withstanding voltage of SMCs [20].

For the phosphoric acid passivation of magnetic particles, the solvents for phosphating used by most factories and researchers are organic solvents, such as ethanol, acetone, and toluene [21,22,23]. However, these solvents greatly limit the ionization of phosphoric acid molecules. The volatilization of organic solvents may cause serious environmental problems. Deionized water, as an environmentally friendly solvent, cannot only reduce environmental pollution but also promote the ionization reaction of phosphoric acid [24]. The effects of different solvents for phosphating on the properties of insulation coating and SMCs have not been studied in detail so far. In this work, deionized water was proposed as the phosphating solvent. The effect of different solvents for phosphating, including deionized water, ethanol, and acetone, on the structure and magnetic properties of phosphated powder and FeSiCr-based SMCs were investigated. The effects of phosphoric acid concentration and temperature on the magnetic properties of FeSiCr SMCs were further studied. In addition, the anti-corrosion properties of the FeSiCr SMCs in different solvents, phosphoric acid concentrations, and temperatures were compared according to the corrosion resistance test.

## 2. Materials and Methods

The water-atomized FeSiCr alloy powder used in this study was provided by Antai Technology Co., Ltd., Beijing, China. The powder has a composition of 87.9–89.3 wt.% Fe, 6.0–6.8 wt.% Si, and 4.7–5.3 wt.% Cr and the average particle size is 10.75 μm. Three different solvents, including ethanol (Et, ≥99.7%, Shanghai Runjie Chemical Reagent Factory, Shanghai, China), acetone (Ac, ≥99.5%, Guangzhou Chemical Reagent Factory, Guangzhou, China), and deionized water (DI) with the same volume, were used in this work. Between 0.1 and 0.5 wt.% phosphoric acid (H_3_PO_4_, ≥85%, Guangzhou Chemical Reagent Factory) was added into Et, Ac, and DI to form homogeneous phosphating solutions, named H_3_PO_4_-Et, H_3_PO_4_-Ac, and H_3_PO_4_-DI, respectively. The FeSiCr alloy powder was phosphated by the prepared solutions for nearly 30 min at different temperatures of 25–65 °C. The phosphating solution suffered a rapid agitation until the solution became slurries. In the case of the solvent with a slow volatilization, such as deionized water, the residual solvent could be removed through the filtration device. It was necessary to disperse the as-phosphated powder through 120 meshes (125 μm) in order to prevent the large agglomeration between them. After that, the sieved powder was completely dried at 120 °C for 30 min in the vacuum drying chamber, and the FeSiCr alloy powder coated with phosphates was obtained. The organic coating solution was prepared by dissolving 0.5 wt.% silane coupling agent (KH550, Guangzhou Fengyu Chemical Co., Ltd., Guangzhou, China), 0.5 wt.% curing agent (diamino diphenyl sulfone, Guangzhou Tiantai New Materials Co., Ltd., Guangzhou, China), and 3.0 wt.% self-made modified silicon polymer resin [25] in acetone. The previously obtained powder was dipped into the organic coating solution and stirred at room temperature until the acetone solvent evaporated completely. In order to obtain the powder with good flowability, the treated powder was passed through a 40–200 mesh screen for the granulation. The composite powder was then obtained and dried at 80 °C for 30 min. Subsequently, the composite powder was mixed with 0.5 wt.% lubricant barium stearate (analytical pure, Mclean Chemical Reagent Co., Ltd., Shanghai, China) and uniaxially pressed into a ring powder core with an outer diameter of 20 mm and an inner diameter of 12 mm under pressures up 600 MPa for 7.5 s. The produced samples were cured at 180 °C for 120 min. In our work, at least three samples were prepared for each group and then tested for their magnetic properties.

The phase identification of raw powders and phosphated powder were analyzed by X-ray diffraction (XRD PANalytical Philips X’ Pert, Almelo, Netherlands) using Cu Kα radiation. The surface morphologies of the raw materials and phosphated powder were characterized by scanning electron microscope (SEM) coupled with an energy dispersive analyzer (EDS) (Nova Nano SEM 430, FEI, Hillsboro, OR, USA). X-ray photoelectron spectrometer (XPS Escalan Xi^+^, Thermo Fisher Scientific, Waltham, MA, USA) was used to investigate the composition and chemical state of the phosphate coating. The saturation magnetization was measured by the Physical Property Measurement System (PPMS-9, Quantum Design, San Diego, CA, USA) equipped with a vibrating sample magnetometer (VSM). The anti-corrosion property of the annular samples was evaluated according to the corrosion resistance test. The withstanding voltage was tested by the AC/DC voltage insulation tester (TH9310, Changzhou Tonghui Electronic Co., Ltd., Changzhou, China). The core loss of the samples was measured by a soft magnetic alternating current test system (MATS-3010SA, Hunan Linkjoin Technology Co., Ltd., Hunan, China). The effective permeability *μ_e_* and quality factor *Q* of the ring magnetic cores were tested by a computer-controlled impedance analyzer (E4990A, Agilent Technologies, Inc., Santa Clara, CA, USA) at low flux densities.

## 3. Results

### 3.1. Effects of Solvent on the Microstructure and Magnetic Properties of Phosphated Powder and SMCs

**Figure 1:**
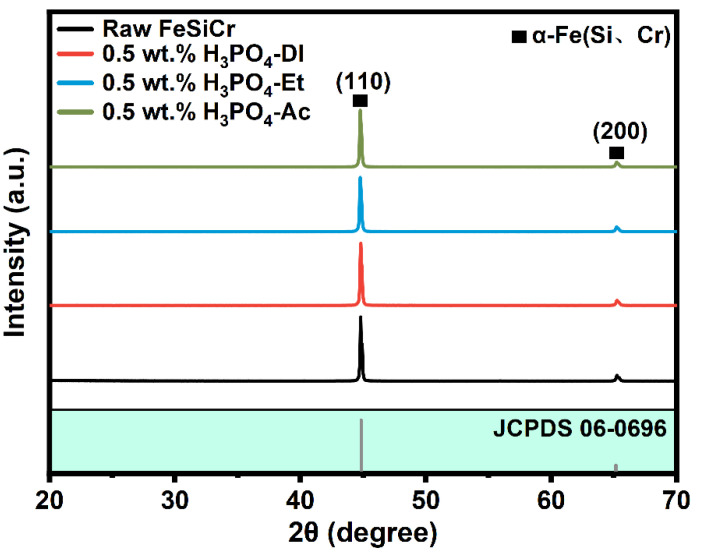
X-ray diffraction patterns of raw powder and phosphated powder prepared in three main solvents.

Figure 1 shows the X-ray diffraction patterns of raw powder and phosphated powder prepared in three main solvents. As shown in Figure 1, the FeSiCr raw powder and phosphated powder treated in three main solvents show three obvious crystallization diffraction peaks at 44.6°and 65.2°, corresponding to (110) and (200) planes of α-Fe(Si, Cr) (PDF #JCPDS 06-0696), respectively. Similar to the results of other workers, the XRD pattern of the phosphated powder failed to detect the characteristic peaks of the phosphate material, which is presumed to be mainly due to the following reasons: (1) The phosphate layer obtained through the phosphating treatment is very thin, which is undetectable by XRD. (2) The phosphate layer may be an amorphous structure and its diffraction signal is covered by the background [21].

**Figure 2:**
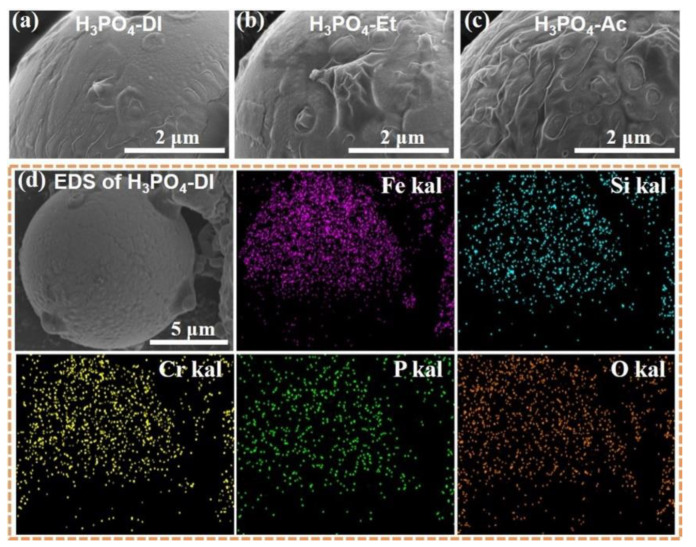
SEM images of phosphated FeSiCr particle prepared in different solvents: (**a**) H_3_PO_4_-DI, (**b**) H_3_PO_4_-Et, (**c**) H_3_PO_4_-Ac, (**d**) the EDS elemental distribution maps of (**a**).

Figure 2 shows the SEM images of phosphated particles prepared in different solvents. The coating on the surface of the FeSiCr particle phosphated in acid-deionized water solution is dense and uniform (Figure 2a), but there are a small number of localized bumps and undulations on the surface of the particle phosphated in the acid–ethanol solution (Figure 2b). Since phosphoric acid is rarely ionized in acetone [26], the coating on the surface of particles phosphated in acid–acetone solution is uneven, resulting in a net-like morphology (Figure 2c) with reduced uniformity. The elemental mapping by EDS of the particle phosphated in acid-deionized water solution is shown in Figure 2d. P and O elements are evenly distributed on the surface of the particle, indicating the formation of uniform phosphate coating.

**Figure 3:**
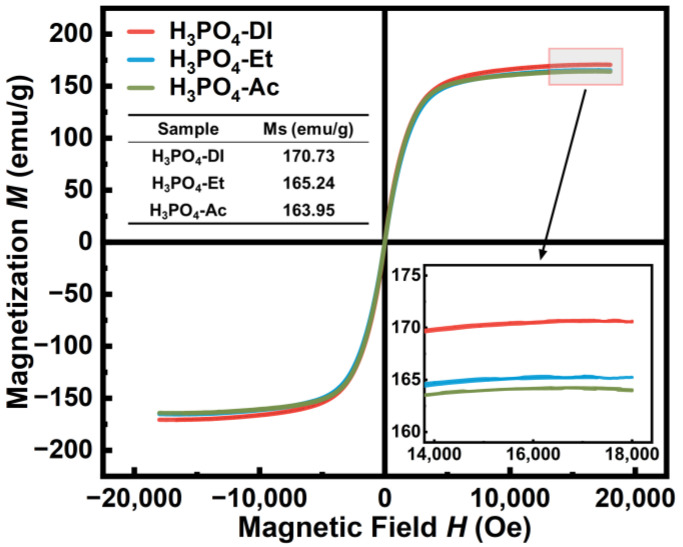
The hysteresis loops of phosphated FeSiCr powder prepared in different solvents.

Figure 3 shows the magnetic hysteresis loops of FeSiCr alloy powder phosphated in different solvents. The enlarged image illustrates the saturation magnetization *M_s_*. The *M_s_* is linearly correlated with the sample density, with high sample density resulting in high *M_s_*. At the same phosphoric acid content of 0.5 wt.%, the density of the FeSiCr powder phosphated in deionized water is maximum 5.47 g/cm^3^, which corresponded to the highest *M_s_* value of 170.73 emu/g. The *M_s_* is reduced by 3.22% to 165.24 emu/g when using the ethanol solution. The FeSiCr powder phosphated in acetone solvent has the lowest *M_s_* value of 163.95 emu/g and also has the lowest density of 5.34 g/cm^3^. Since the reduced *M_s_* results from the formation of non-magnetic phosphate layer [27], the result indicates that FeSiCr powder phosphated in deionized water has a thinner phosphate coating compared to the organic solvents. Hence, deionized water is beneficial to maintaining the high-saturation properties of the SMCs.

**Figure 4:**
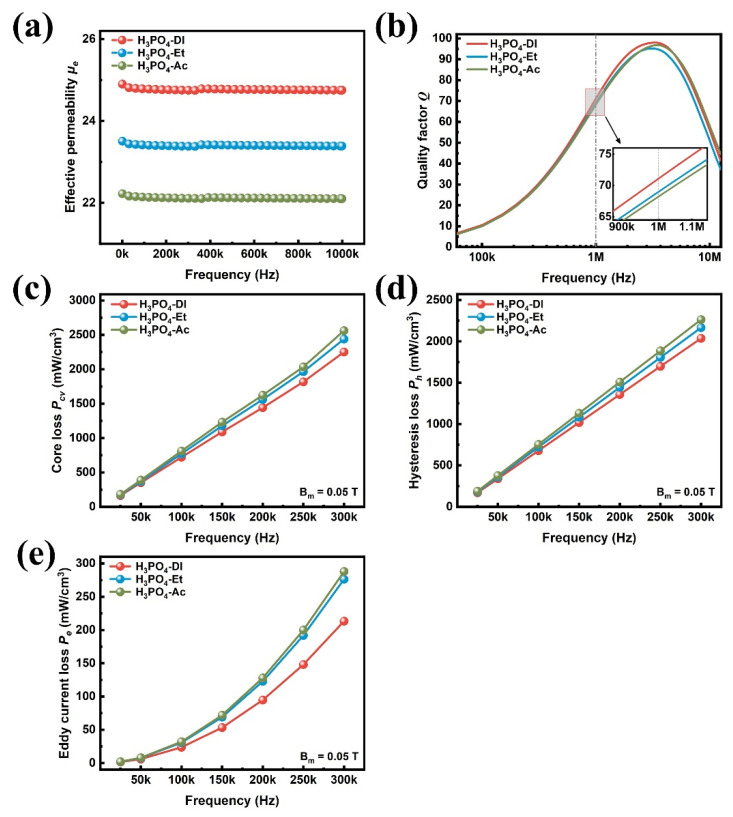
Magnetic performances of FeSiCr SMCs prepared by phosphating in different solvents: (**a**) effective permeability *µ_e_*, (**b**) quality factor *Q*, (**c**) core loss *P_cv_*, (**d**) hysteresis loss *P_h_*_,_, and (**e**) eddy current loss *P_e_* versus frequency at 0.05 T.

Figure 4 shows the magnetic property variations with frequency for the FeSiCr SMCs prepared in different phosphating solvents. In detail, Figure 4a shows the variation of effective permeability (*μ_e_*) for all samples from 1 kHz to 1 MHz. The three solvents exhibit excellent frequency stability of *µ_e_* up to 1 MHz. Usually, the higher the density of the sample, the higher the permeability. At 1 MHz, the highest *µ_e_* of 24.7 is obtained in deionized water, 1.3, and 2.6 higher than in ethanol and acetone, respectively. Similarly, as shown in Figure 4b, the *Q* in deionized water is the highest at 71.6, 3.0%, and 3.6% higher than in ethanol and acetone, respectively. Figure 4c gives the total experimental loss *P_cv_* for all SMCs at frequencies ranging from 25 kHz to 300 kHz and the magnetic flux density (Bm = 0.05 T). The SMCs phosphated in deionized water obtained the lowest core loss of 759.4 mW/cm^3^ measured at 0.05 T and 100 kHz. In comparison, the core loss of SMCs phosphated in ethanol and acetone is 771.0 mW/cm^3^ and 811.9 mW/cm^3^, with 6.47% and 11.18% increases, respectively.

Figure 4d,e show the frequency dependences of the hysteresis losses and the eddy current losses for all samples. The hysteresis losses linearly increase with frequency, as shown in Figure 4d. In addition, Figure 4e shows that the eddy current losses increase quadratically with frequency. Compared with the samples treated with acid–acetone and acid–ethanol solution, the samples coated with acid-deionized water solution show decreased hysteresis losses, as shown in Figure 4d. The surface of particles phosphated in deionized water was smoother and more complete than in acetone and ethanol. Therefore, the demagnetization field formed by non-magnetic materials and gaps is small, reducing the coercive force and the hysteresis loss [28]. Notably, the eddy current losses of the samples phosphated in deionized water decrease dramatically, as shown in Figure 4e. The eddy current induces eddy current loss under high frequency. The phosphate coating on the surface of the coated particles can effectively reduce the eddy current loss [29]. Therefore, the phosphate coating in deionized water becomes dense and even, and the eddy current loss becomes smaller, which exhibits the best insulating properties. The detailed values of the effective permeability *µ_e_*, quality factor *Q*, and core loss *P_cv_* at selected frequencies and the withstanding voltage are summarized in Table 1. Withstanding the voltage indicates the ability to withstand large voltage loads for SMCs working under AC mode operation. It is measured in AC mode. The outputs and inputs are connected to the sample by means of conductive cable cleats, and the control terminal sets parameters such as voltage rise and hold times. The external electric field is continuously increased until the SMC sample is penetrated, and the withstanding voltage is recorded at the current leakage threshold of 1 mA. Raw powder can be regarded as a metallic conductor with very low withstand voltage. After insulation coating, the resistivity of the material increases, resulting in a higher withstand voltage. The high voltage also indicates the coating effectiveness of the phosphate layer. The results show that SMCs prepared in deionized water have higher electromagnetic properties, such as a higher withstanding voltage, higher *μ_e_*, higher *Q,* and lower *P_cv,_* than ethanol and acetone.

**Figure 5:**
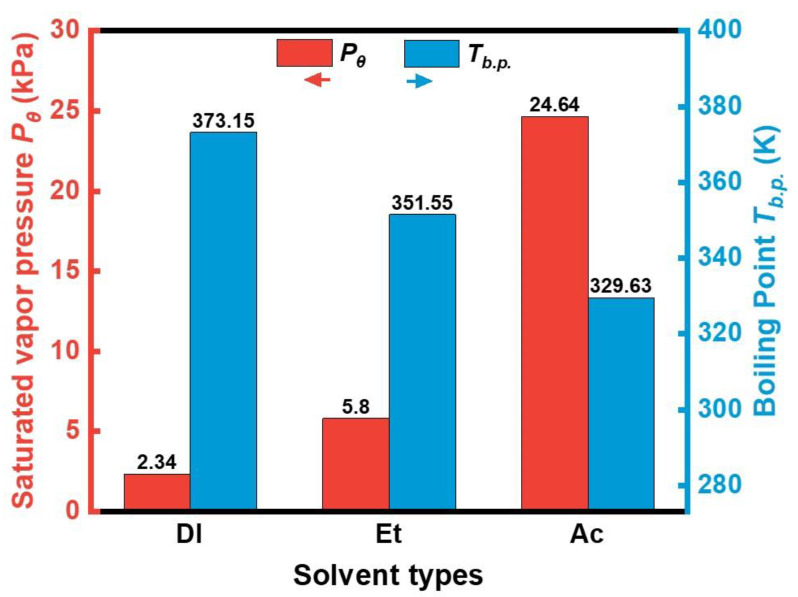
Differences in physical properties for different solvents.

**Figure 6:**
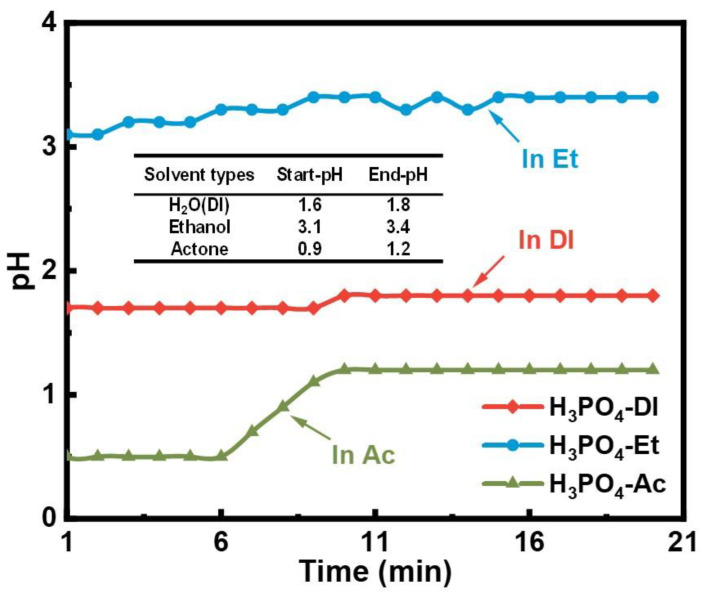
The relationship between the pH value and reaction time in different solvents.

Figure 5 shows the differences in physical properties of the three solvents. The saturation vapor pressure of the solvent is negatively correlated with the boiling point. Water has the lowest saturation vapor pressure of 2.3 kPa (20 °C), much lower than ethanol and acetone. Therefore, in the temperature below the boiling point range, the volatilization rate of water is the lowest. The phosphating process is a wet chemical treatment [30]. When the particles are phosphated in deionized water solvent, the volume of the solution can be stabilized. The relatively stable solution volume allows for a more accessible and controllable phosphating process. However, in the other two preparation processes, ethanol and acetone evaporate due to their high saturated vapor pressure, and the volume of the phosphating solution gradually decreases. It fails to completely submerge the powder in severe cases, resulting in an aborted phosphating reaction. The instability of the volume of the phosphating solution also led to the drastic fluctuation of the solution pH with reaction time in ethanol and acetone. Figure 6 shows the pH variation with time for different phosphating solutions. As shown in Figure 6, the phosphating reaction is carried out under acidic conditions (pH < 7.0). The pH value increases with increasing time. During the phosphating process, the metal atoms released electrons to reduce the hydroxide ions to hydrogen, resulting in a large consumption of hydrogen ions, and when the reaction was carried out for 20 min, the pH values of all three phosphoric acid solutions increased by 0.2–0.3. However, the pH–time curve for acid-deionized water solution was relatively smooth compared to the acid–ethanol and acid–acetone solution. On the one hand, the volumetric instability of the phosphating solution in ethanol and acetone leads to drastic fluctuations in the solution pH with reaction time. On the other hand, phosphoric acid molecules are more likely to undergo tertiary ionization in aqueous solutions to form stable trivalent ferric phosphate products. In contrast, in ethanol and acetone solutions, the process of phosphoric acid ionization is incomplete, and sub-stable monohydrogen phosphate products are formed. The conversion of sub-stable monohydrogen phosphate salts into stable phosphate salts may affect the overall stability of the phosphoric acid solution. Therefore, the phosphating reaction in deionized water is more stable than in acetone and ethanol, and a complete and even phosphate film can be obtained.

**Figure 7:**
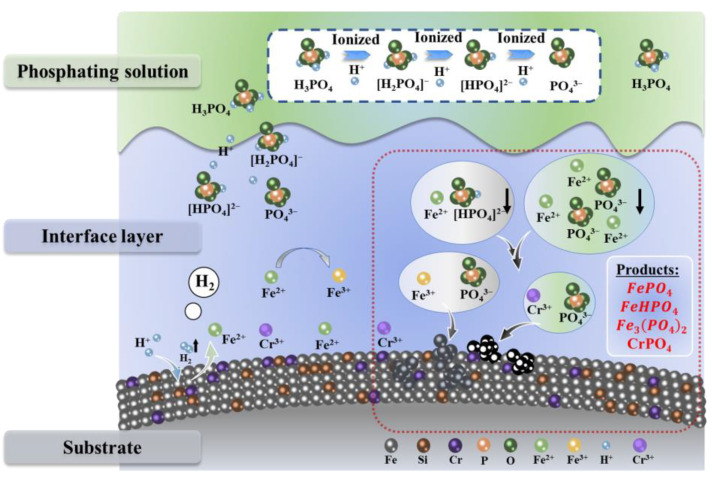
Schematic diagram of the formation of phosphate coating.

The mechanism of the formation of the phosphate layer is schematically shown in Figure 7. The reactions that occurred at the surface of the FeSiCr powder are described by Equations (1)–(6). Unlike iron powder, FeSiCr tends to form a dense Cr-O layer on the particle surface due to the presence of the Cr element, which is the main reason for the excellent aging resistance of FeSiCr SMCs [31]. Consequently, the reaction is preceded by the dissolution of the oxide film on the surface of the FeSiCr powder (Equation (1)). When the FeSiCr powder is immersed in the H_3_PO_4_ solution, Fe atoms are rapidly ionized to form Fe^2+^ ions, as shown in Figure 7. The electrons generated by the reaction are transferred to the H^+^ ions of H_3_PO_4_ ionization, and then, the hydrogen gas is released (Equation (2)) [32,33]. The formation of hydrogen increases the pH value at the particle/solution interface, which then promotes the tertiary ionization of H_3_PO_4_ and generates more PO_4_^3-^ ions (Equation (3)). The Fe^2+^ ions are enriched on the particle/solution interface, resulting in the rapid formation and deposition of ferrous phosphate on the surface of the particle (Equation (4)). In addition, the Fe^2+^ ions may be oxidized to Fe^3+^ ions, and then iron phosphate with PO_4_^3-^ ions may be formed (Equations (5) and (6)).

Cr_2_O_3_ + 2H_3_PO_4_ → 2CrPO_4_↓ + 3H_2_O (1)

Fe + 2H_3_PO_4_ → Fe(H_2_PO_4_)_2_ + H_2_↑ (2)

H_3_PO_4_ → H^+^ + H_2_PO_4_^−^ → 2H^+^ + HPO_4_^2−^ → 3H^+^ + PO_4_^3−^ (3)

3Fe(H_2_PO_4_)_2_ ↔ 3FeHPO_4_↓ ↔ Fe_3_(PO_4_)_2_↓ (4)

4Fe(H_2_PO_4_)_2_ + O_2_ → FePO_4_↓ + 4H_3_PO_4_ + H_2_O (5)

FeHPO_4_ + Fe_3_(PO_4_)_2_ + H_3_PO_4_ + O_2_ → 4FePO_4_↓ + 2H_2_O (6)

**Figure 8:**
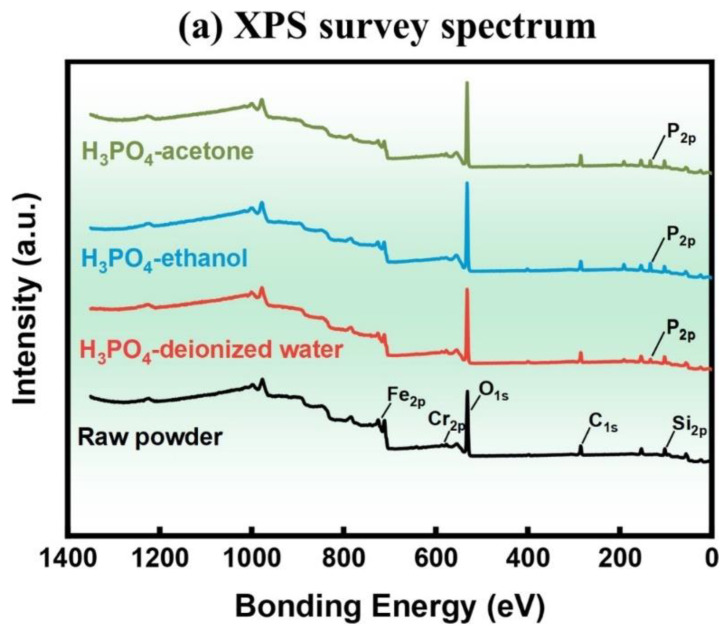

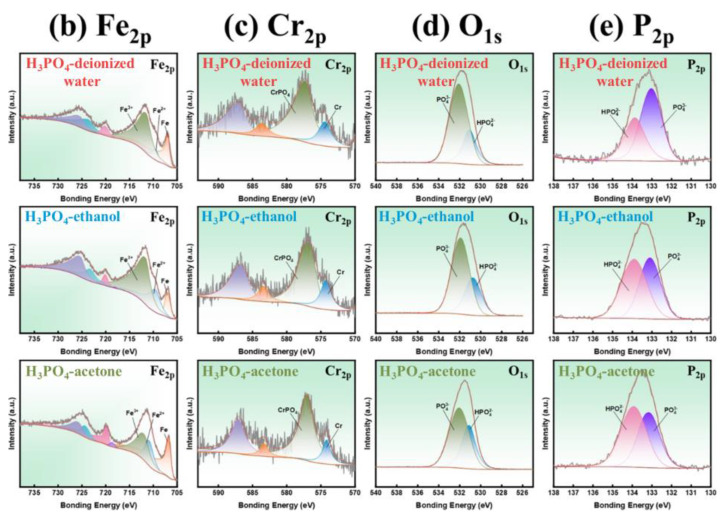
XPS spectra of phosphated powder prepared in different solvents: (**a**) XPS survey spectrum, (**b**) Fe_2p_, (**c**) Cr_2p_, (**d**) O_1s,_ and (**e**) P_2p_ peaks.

Figure 8a shows the survey XPS spectra of the phosphated powder prepared in different solvents, and the detailed XPS spectra of Fe_2p_, Cr_2p_, O_1s_, and P_2p_ peaks are shown in Figure 8b–e, respectively. The Fe_2p_ spectra contain Fe (706.8 eV), Fe^2+^ (711.0 eV), and Fe^3+^ (712.0 eV) peaks [34,35]. In Cr_2p_ spectra, Cr (574.2 eV) and CrPO_4_ (577.1 eV) are detected [32,36,37]. The O_1s_ peaks can be fitted at 530.7 eV and 531.9 eV, corresponding to HPO_4_^2−^ and PO_4_^3−^, respectively [37,38]. The P_2p_ peaks can be fitted with 2p_1/2_ and 2p_3/2_ components at 133.9 eV and 133.0 eV, corresponding to HPO_4_^2-^ and PO_4_^3−^, respectively [39,40]. These results demonstrate that the phosphate coating of the FeSiCr-based powder mainly comprises iron phosphates with Cr^3+^, Fe^2+^, Fe^3+^, PO_4_^3−^, and HPO_4_^2−^ ions. Fe_3_(PO_4_)_2_, FeHPO_4_, FePO_4,_ and CrPO_4_ may also be present in the phosphates coating [41]. The result of the fitted split peak area shows that the product of phosphorylation in deionized water contains more Fe^3+^ ions and PO_4_^3−^ ions, which indicates that phosphoric acid is more ionizable in deionized water, allowing more PO_4_^3-^ ions to combine with Fe^3+^ ions to form FePO_4_. Furthermore, the deionized water contains a higher level of dissolved oxygen, and the Fe^2+^ phosphate is more readily oxidized into the more stable Fe^3+^ phosphate, resulting in a more stable phosphate layer structure.

### 3.2. Process Optimization for SMCs Prepared in Deionized Water

**Figure 9:**
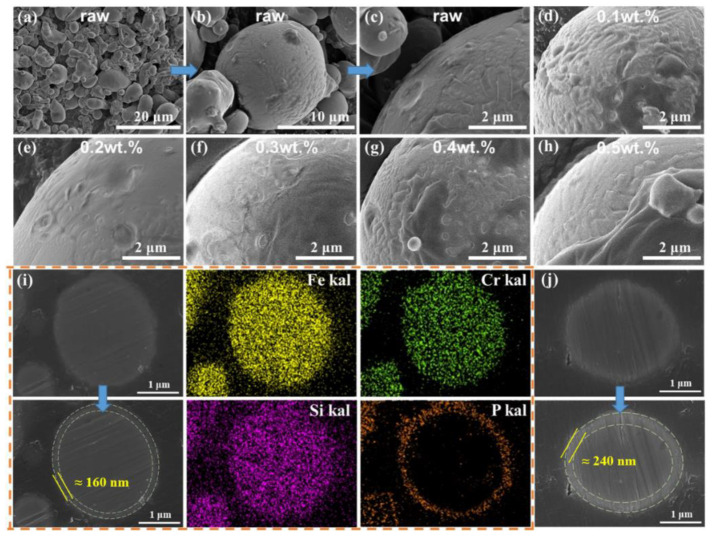
SEM images of FeSiCr particle phosphated by different phosphoric acid concentrations of the H_3_PO_4_-DI solution: (**a**–**c**) raw powder particles under different magnification, (**d**) 0.1 wt.%, (**e**) 0.2 wt.%, (**f**) 0.3 wt.%, (**g**) 0.4 wt.%, (**h**) 0.5 wt.%, (**i**) cross-section and the EDS elemental distribution maps of particles of (**e**), (**j**) cross-section of particles of (**h**).

To further improve the properties of SMCs prepared in DI solvent, the phosphating process was optimized by adjusting the concentration and temperature of the solution. The SEM and EDS spectra of the phosphated powder prepared with a different phosphoric acid concentration in DI are demonstrated in Figure 9. The morphologies of the raw powder surface are shown in Figure 9a–c. There are some liquid-flow streaks on the surface of water atomized raw powder due to high-pressure water jets for rapid cooling. After phosphating, the streaks disappeared gradually as the phosphate products were preferentially deposited in the streak pits on the powder surface. However, an increase in the phosphoric acid content leads to an increase in powder surface roughness, as shown in Figure 9d–h. According to the EDS results, the surface layer consists of iron (Fe), silicon (Si), chromium (Cr), and phosphorus (P) elements after phosphating, indicating a phosphate layer was formed successfully. We can note that there is a brightness difference between the outer shell layer and the inner core of the powder particles (Figure 9i,j), which is caused by the difference in the scattering of electrons due to the different structures and compositions of the inner and outer layers. Based on the distribution of the corresponding P elements, we surmise that the outer edge layer is the phosphate coating. The thickness of the phosphate coating was estimated to be about 160–240 nm when the phosphoric acid concentration was in the range of 0.2–0.5 wt.%. Thus, the relationship between phosphoric acid concentration on the thickness of the outer shell layer can be visualized by measuring the thickness of the phosphate coating layer. This result indicates that the thickness of phosphate coating can be controlled by the concentration of phosphoric acid. The phosphate coating formed on the surface of the particles is relatively thin and dense using the present process.

**Figure 10:**
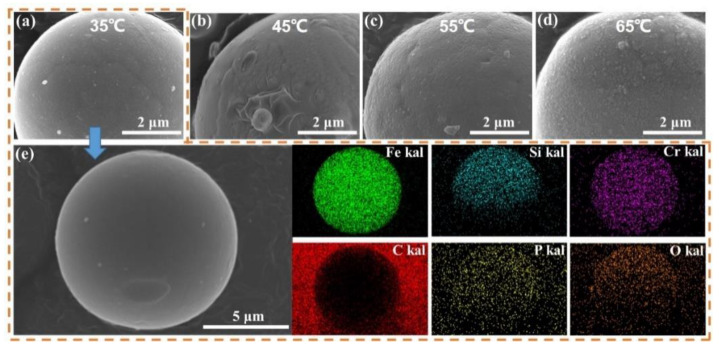
SEM images of FeSiCr particle phosphated at different temperatures of H_3_PO_4_-DI solution: (**a**) 35 °C, (**b**) 45 °C, (**c**) 55 °C, (**d**) 65 °C, (**e**) the EDS elemental distribution maps of (**a**).

Figure 10a–d illustrates the surface morphology of phosphate coating on the particle at different phosphating temperatures of 35 °C, 45 °C, 55 °C, and 65 °C. As the temperature increases, the surface of the coated particle becomes rougher. As we know, high temperatures can promote the rapid nucleation and growth of phosphate crystals. However, excessively high temperatures result in the precipitation of coarse island-shaped phosphate on the particle surface [24,32]. Figure 10e displays the surface morphology of the phosphated particle obtained at 35 °C. Along with the EDS results, it can be observed that phosphorus (P) is mainly concentrated on the outer surface of the particle and is uniformly distributed, which further confirms the presence of the phosphate coating.

**Figure 11:**
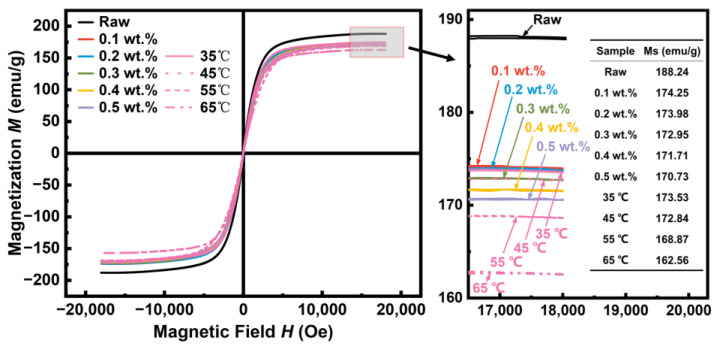
The hysteresis loops and enlarged view of the identified area of FeSiCr alloy powder phosphated by different phosphoric acid concentrations and temperatures of H_3_PO_4_-DI solution.

The magnetic hysteresis loops of the FeSiCr raw powder and the coated powder phosphated by different concentrations and at various temperatures are shown in Figure 11. All samples exhibit typical soft magnetic characteristics. The *M_s_* for the powder prepared with 0.1 wt.% H_3_PO_4_-DI solution is 174.25 emu/g, decreased by 7.43%, compared to 188.24 emu/g for the initial raw powder. *M_s_* continues to decrease to 170.73 emu/g with increasing H_3_PO_4_ concentration to 0.5 wt.%. The monotonically decreased *M_s_* with increasing H_3_PO_4_ phosphating concentration and temperature results from more non-magnetic phosphating products being produced as the acid concentration and temperature increase.

**Figure 12:**
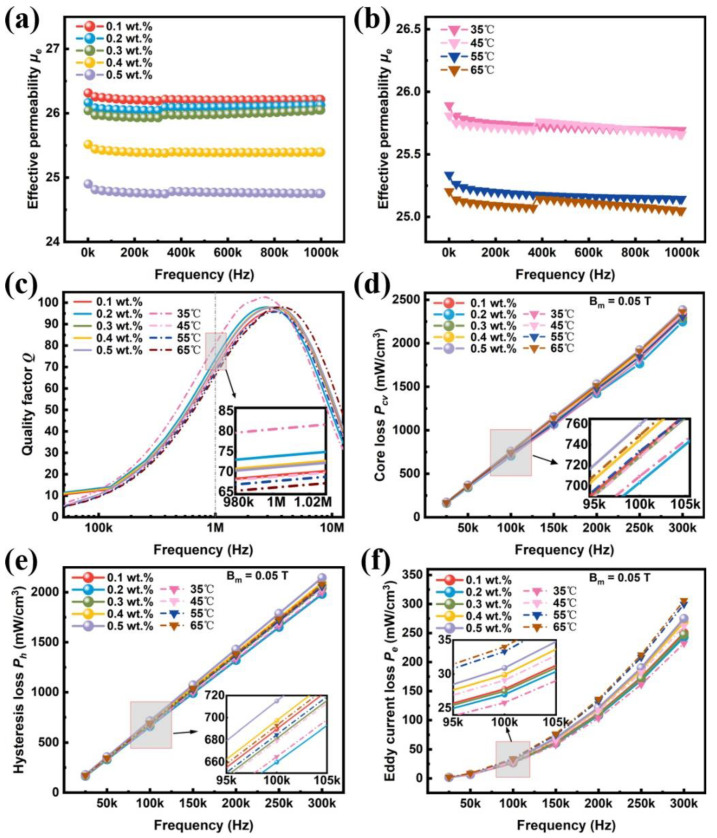
Magnetic performance of FeSiCr SMCs prepared with phosphate in different phosphoric acid concentrations and different temperatures of H_3_PO_4_-DI solution: (**a**) effective permeability *µ_e_* for phosphoric acid concentration; (**b**) effective permeability *µ_e_* for temperature; (**c**) quality factor *Q*; (**d**) core loss *P_cv_*; (**e**) hysteresis loss *P_h_*; and (**f**) eddy current loss *P_e_* versus frequency at 0.05 T.

The variations of the effective permeability *µ_e_*, quality factor *Q*, and core loss *P_cv_* of FeSiCr SMCs prepared with phosphated powder in a 0.1–0.5 wt.% concentration at 25–65 °C are shown in Figure 12. All samples show a good frequency stability up to 1 MHz. As shown in Figure 12a, with the increase in the phosphoric acid concentration from 0.1 wt.% to 0.5 wt.%, *µ_e_* decreased from 26.2 to 24.7 due to the increase in the non-magnetic insulating phosphate layer coated on the particles. With the temperature rise from 35 °C to 65 °C, *µ_e_* slightly decreases from 25.7 to 25.0. In Figure 12b, the SMCs prepared by coating with 0.2 wt.% phosphoric acid solution at 35 °C show the highest *Q* of 80.2 at a frequency of 1 MHz, indicating the excellent high-frequency characteristics of the FeSiCr SMCs. With the increase in the phosphoric acid concentration, the frequency corresponding to the highest *Q* peak of the sample shifts to higher frequency, which is favorable for high-frequency applications. With the increase in the temperature, the value of *Q* increases first due to the formation of dense insulating phosphate coating. Then, it decreases gradually with a further increase in the non-magnetic and inhomogeneous island-shaped phosphate.

In order to analyze the phosphating effect on the total core loss (*P_cv_*), loss separation is performed. The residual loss (*P_r_*) is significant only at very low induction levels and high frequency, but it can usually be ignored in applications. Hence, the total core loss (*P_cv_*) can be presented as follows [7,42,43]:

(7) $$P_{cv}=P_{h}+P_{e}+P_{r}\approx P_{h}+P_{e}=C_{h}\times f+C_{e}\times f^{2}$$

where *C_h_* and *C_e_* are the coefficients for the hysteresis loss (*P_h_*) and eddy current loss (*P_e_*), and *f* is the frequency. It can be seen that the total core loss *P_cv_* is a function of frequency, with the hysteresis loss *P_h_* linearly correlated with frequency and the eddy current loss *P_e_* quadratically correlated with frequency. The *P_cv_* is the measured total loss, fitted a binomial to the *P_cv_*(*f*) curve to obtain the quadratic term coefficient *C_e_* and the primary term coefficient *C_h_*, which were taken into Equation “*P_e_ = C_e_* × *f*^2^” and “*P_e_ = C_e_* × *f*” to obtain the fitted eddy current loss *P_e_* and hysteresis loss *P_h_*, respectively. The residual loss *P_r_* is then obtained by subtracting the hysteresis loss and eddy current loss from the total core loss *P_cv_*. Figure 12c gives the total core loss *P_cv_* for all samples at frequencies ranging from 25 to 300 kHz under a magnetic flux density *B_m_* of 0.05 T. The sample prepared with 0.2 wt.% phosphoric acid solution at 25 °C exhibits a much lower *P_cv_* of 703.5 mW/cm^3^ compared with the other samples. The separated *P_h_* and *P_e_* are shown in Figure 12d,e. It is clear that *P_h_* is dominant at a low frequency of 25–300 kHz, and it accounts for more than 90% of the total loss at 100 kHz.

**Figure 13:**
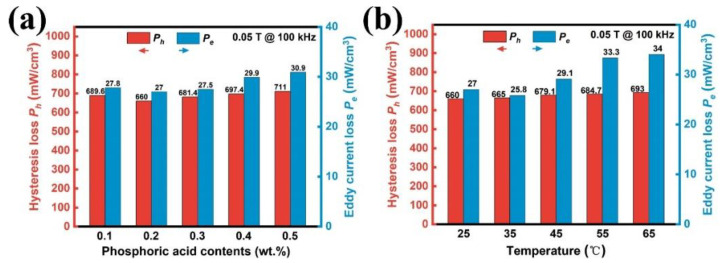
The contribution of the *P_h_* and the *P_e_* for the SMCs samples, which were fitted under 100 kHz and 0.05 T (**a**) SMCs phosphated in different phosphoric acid concentrations and (**b**) SMCs phosphated at different temperatures.

Figure 13 shows the loss separation of *P_cv_* at 100 kHz and *B_m_* = 0.05 T. With the increase in phosphoric acid concentration and temperature, the hysteresis loss *P_h_* exhibits an increasing trend. The eddy current loss *P_e_* decreases first and increases afterward. For phosphoric acid concentrations of 0.1–0.5 wt.%, the *P_e_* reaches its lowest peak at 0.2 wt.%. For temperatures ranging from 25 °C to 65 °C, the lowest *P_e_* of 25.8 mW/cm^3^ occurs at 35 °C, indicating that that a complete phosphate coating has been obtained on the magnetic particle surface. The minimum core loss *P_cv_* of 703.5 mW/cm^3^ at 100 kHz is obtained for the phosphated SMCs prepared with 0.2 wt.% phosphoric acid at 35 °C. Compared with the other SMCs samples, the highest quality factor *Q* of 80.2 at 1 MHz is obtained for the SMCs phosphated with 0.2 wt.% acid solution at 35 °C. The highest quality factor also indicates that the phosphate coating has the best insulation effect. The detailed values of the effective permeability *µ_e_*, quality factor *Q*, core loss *P_cv_,* and the withstanding voltage are summarized in Table 2.

Table 3 lists the soft magnetic properties of the SMCs obtained in this study and those reported in recent years. The results indicate that the SMCs phosphated in deionized water in this study have excellent soft magnetic properties, especially higher permeability *μ_e_* and lower total core loss *P_cv_*, as previously reported. The results suggest that the phosphating effect of the powder phosphated in deionized water is better than those of ethanol and acetone, resulting in the dense phosphate coating on the particle surface and excellent soft magnetic properties of the SMCs. Therefore, deionized water is highly proposed as an environmentally friendly phosphating solvent.

### 3.3. Corrosion Resistance

Table 4 shows the corrosion resistance of raw SMCs and phosphated SMCs prepared by different phosphating processes. The corrosion area can assess the quality of the phosphate-insulating coating on the particle surface under 5.0% NaCl solution at 35 °C. The results show that raw SMCs have an inferior corrosion resistance since the corrosive solution is directly in contact with the substrate. The corrosion resistance of the phosphated SMCs prepared by phosphated powder has been significantly improved compared to raw SMCs. For different phosphating solvents, the corrosion resistance of the phosphated SMCs using deionized water as a phosphating solvent is better than that of using ethanol and acetone. This is because the dense phosphate coating on the surface of the particle effectively prevents contact between the corrosive medium and the substrate, slowing down the corrosion rate. Moreover, different phosphoric acid concentrations and temperatures can affect the quality of the coating of particles to a certain extent, resulting in differences in corrosion resistance.

## 4. Conclusions

In this work, the effect of different solvents for phosphating, including deionized water, ethanol, and acetone, on the structure and magnetic properties of FeSiCr-based SMCs were investigated. It was found that the phosphating reaction in deionized water is more stable in deionized water, resulting in dense phosphate coating on the particle surface. The phosphate product in deionized water contains more trivalent iron phosphate than divalent ferrous phosphate. For deionized water, the SMCs exhibit the best insulating properties and excellent magnetic performances due to a more complete and thinner coating layer. Moreover, the effects of phosphoric acid concentration and temperature on the magnetic properties of FeSiCr SMCs prepared in deionized water were further studied. With the increase in phosphoric acid concentration and temperature, the magnetic permeability and saturation magnetization of the powder core decrease, and the core loss decreases firstly, followed by an increase. The optimized combination of properties was obtained for the SMCs phosphated with 0.2 wt.% phosphoric acid in deionized water at 35 °C, including high effective permeability *μ_e_* of 25.7, high quality factor *Q* of 80.2, low core loss *P_cv_* of 709.5 mW/cm^3^ measured at 0.05 T @ 100 kHz, and high withstanding voltage of 276 V, due to the formation of more uniform and dense insulating coating layers. In addition, the SMCs prepared with phosphated powder show good corrosion resistance. The corrosion resistance of the phosphated SMCs using deionized water as a phosphating solvent is better than that of ethanol and acetone. Hence, deionized water can be proposed as an environmentally friendly phosphating solvent.

## Figures and Tables

**Table 1:** Properties of FeSiCr SMCs prepared in different phosphating solvents.

Sample	Density	*µ_e_* (1 MHz)	*Q* (1 MHz)	*P_cv_*/(mW·cm^−3^) @ 0.05 T		Withstanding Voltage (V)
	(g/cm^3^)			100 kHz	200 kHz	
0.5 wt.% H_3_PO_4_-DI	5.47	24.7	71.6	759.4	1533.0	241
0.5 wt.% H_3_PO_4_-Et	5.40	23.4	69.5	771.0	1561.0	225
0.5 wt.% H_3_PO_4_-Ac	5.34	22.1	69.1	811.9	1624.0	217

**Table 2:** Comprehensive performance of FeSiCr SMCs prepared with phosphated powder in different phosphoric acid concentrations and temperatures.

Sample	*µ_e_* (1 MHz)	*Q* (1 MHz)	*P_cv_*/(mW·cm^−3^) @ 0.05 T		Withstanding Voltage (V)
			100 kHz	200 kHz	
0.1 wt.% H_3_PO_4_-DI	26.2	69.4	731.0	1476.0	259
0.2 wt.% H_3_PO_4_-DI	26.1	74.7	703.5	1423.0	269
0.3 wt.% H_3_PO_4_-DI	26.0	72.0	728.4	1461.0	250
0.4 wt.% H_3_PO_4_-DI	25.4	72.5	744.7	1503.0	246
0.5 wt.% H_3_PO_4_-DI	24.7	71.6	759.4	1533.0	241
35 °C @ H_3_PO_4_-DI	25.7	80.2	709.5	1431.0	276
45 °C @ H_3_PO_4_-DI	25.6	68.7	728.1	1463.0	281
55 °C @ H_3_PO_4_-DI	25.1	67.2	733.6	1471.0	287
65 °C @ H_3_PO_4_-DI	25.0	65.5	749.0	1515.0	291

**Table 3:** Comparison of magnetic properties of the SMCs obtained in this study with the typical Fe-based SMCs reported previously.

References	Powder	Phosphating Solvent	Molding Pressure (MPa)	*µ_e_*	*P_cv_*/(mW·cm^−3^) ^a^	
					100 kHz	200 kHz
[22]	CIP	Acetone	580	14.0	--	--
[23]	FeSiCr	Ethanol	600	20.6	--	2087
[44]	FeSiCr	Ethanol	265	16.3	1500	3550
[45]	FeSiCr	Acetone	600	44.5	780	--
This work	FeSiCr	Deionized water	600	25.7	709.5	1431.0

^a^ *P_cv_* is measured at 0.05 T.

**Table 4:** The corrosion resistance of raw SMCs and phosphated SMCs prepared by different phosphating processes (solvents, phosphoric acid concentrations, and temperatures).

Phosphated in different solvents (0.5 wt.% H_3_PO_4_ concentration; 25 °C)	Solvents	Raw	Acetone	Ethanol	Water	/
	Photograph after corrosion resistance test	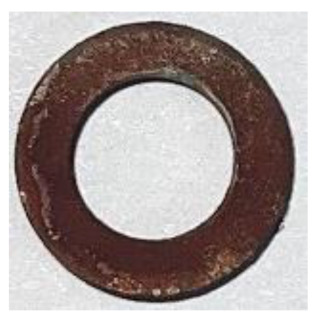	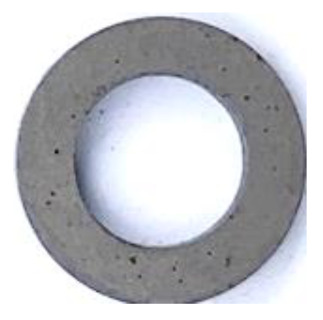	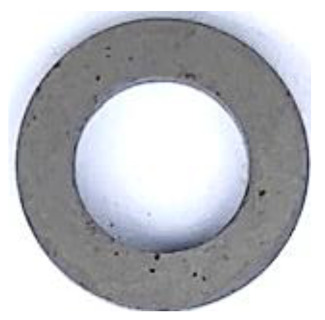	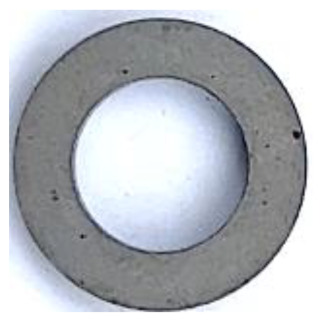	/
	Corrosion area ratio (%)	>96%	<1.0%	<0.5%	<0.3%	/
Phosphated with different H_3_PO_4_ concentrations (in deionized water; 25 °C)	H_3_PO_4_ concentrations	0.1 wt.%	0.2 wt.%	0.3 wt.%	0.4 wt.%	0.5 wt.%
	Photograph after corrosion resistance test	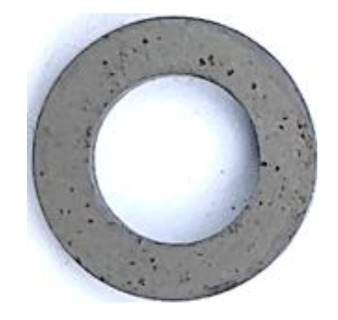	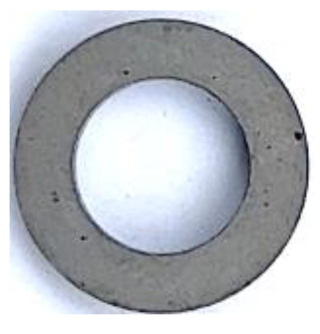	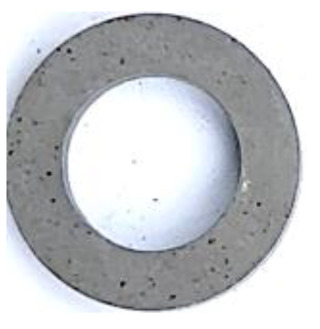	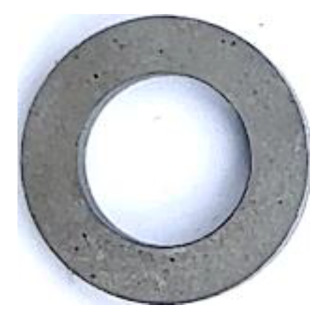	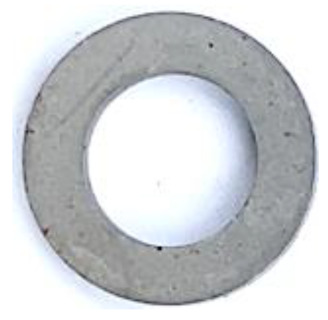
	Corrosion area ratio (%)	<1.0%	<0.3%	<0.5%	<0.5%	<0.1%
Phosphated at different temperatures (in deionized water; 0.2 wt.% H_3_PO_4_ concentration)	Temperatures	25 °C	35 °C	45 °C	55 °C	65 °C
	Photograph after corrosion resistance test	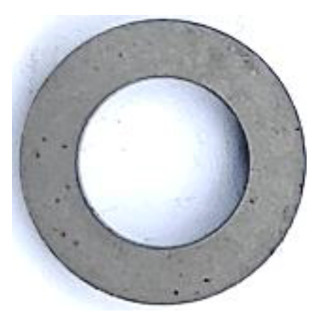	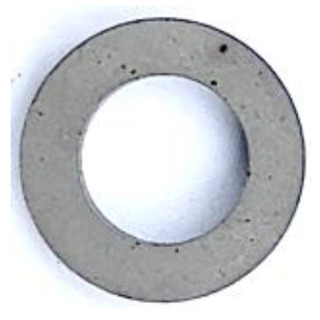	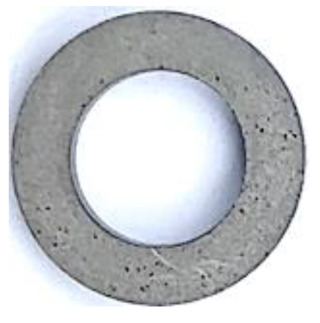	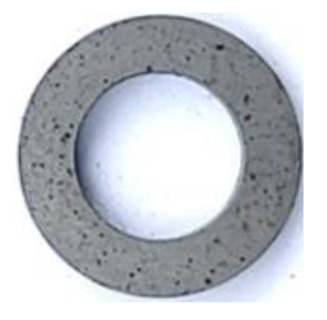	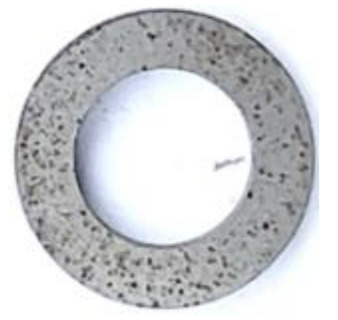
	Corrosion area ratio (%)	<0.3%	<0.1%	<1.0%	<5.0%	<10.0%

Items and test conditions: corrosion resistance test in the conditions of 5.0% NaCl solution, the temperature of 35 °C, pH value of 6.5~7.2, and time of 72 h. (Ref. [46]: ASTM B895−16).

## Data Availability

Seek out the author to get the raw data.
